# Effect of liver abnormalities on mortality in Fontan patients: a systematic review and meta-analysis

**DOI:** 10.1186/s12872-024-04042-3

**Published:** 2024-07-25

**Authors:** Xiang Liu, Linjiang Han, Ziqin Zhou, Jiazichao Tu, Jianrui Ma, Jimei Chen

**Affiliations:** 1 Department of Cardiac Surgery, Guangdong Provincial People’s Hospital (Guangdong Academy of Medical Sciences), Guangdong Cardiovascular Institute, Southern Medical University, No. 106, Zhongshan 2nd Road, Guangzhou, 510080 PR China; 2 Guangdong Provincial Key Laboratory of South China Structural Heart Disease, Guangzhou, 510080 China

**Keywords:** Fontan procedure, Fontan-associated liver disease, Mortality, Transplantation, Meta-analysis

## Abstract

**Background:**

Fontan-associated liver disease (FALD) is one of the most common complications following Fontan procedure, but the impact of FALD on survival outcomes remains controversial. The aim of this systematic review and meta-analysis was to examine and quantify the influence of liver disease on the survival of Fontan patients.

**Methods:**

The Preferred Reporting Items for Systematic reviews and Meta-Analyses guidelines were followed, and relevant human studies published from inception up to 12 August 2022 were searched. Stata (version 17.0) was applied to perform the meta-analysis, using random effects (Mantel-Haenszel) models. The *I*^*2*^ statistic was used to assess the heterogeneity. Subgroup analysis and meta-regression were employed to explore the potential sources of heterogeneity and sensitivity analysis was performed to determine the potential influence of each study on the overall pooled results.

**Results:**

A total of 312 records were initially identified and 8 studies involving 2,466 patients were selected for inclusion. Results revealed a significant association between the severity of liver disease following Fontan procedure and mortality, which was confirmed by sensitivity analysis and subgroup analysis assessing post-HT mortality. Meta-regression showed that diagnostic methods for liver disease may be a source of heterogeneity. After removal of the FALD patients identified by international classification of disease codes, heterogeneity was markedly reduced, and the positive association between all-cause mortality and the severity of liver disease became significant.

**Conclusions:**

This meta-analysis showed the severity of liver disease following the Fontan procedure has a significant association with mortality. Lifelong follow-up is necessary and imaging examinations are recommended for routine surveillance of liver disease. Among patients with failing Fontan and advanced liver disease, combined heart-liver transplantation may provide additional survival benefits.

**Supplementary Information:**

The online version contains supplementary material available at 10.1186/s12872-024-04042-3.

## Background

Since first reported in 1971 [1], Fontan procedure has evolved and now is the gold standard operation for patients with single ventricle physiology. With the great progress in surgical techniques and perioperative managements, the long-term survival of Fontan population has significantly improved over the past few decades [2, 3]. It is estimated that 70,000 patients around the world are currently living with Fontan circulation and this number is expected to double during the next 20 years [4]. However, many patients complicated by multiple organ dysfunction and suffer from a poor quality of life and a substantial increased risk of premature mortality [2].

Fontan-associated liver disease (FALD) is one of the most common complications following Fontan procedure, which is characterized by hepatic fibrosis and even cirrhosis and generally develops slowly and gets worse over time [5]. Despite it is highly prevalent in Fontan patients especially for those who have Fontan procedure more than 10 years [6], the impact of FALD on survival outcomes remains controversial [7–13]. These discrepancies between studies may be caused by single-center experiences and small sample sizes, therefore, it is important to integrate these findings from different studies. The aim of this systematic review and meta-analysis was to examine and quantify the influences of liver disease on mortality in Fontan patients.

## Methods

### Registration

The protocol for this systematic review and meta-analysis was prospectively registered on PROSPERO (CRD42022359113). The Preferred Reporting Items for Systematic reviews and Meta-Analyses (PRISMA) guidelines were followed for the current study [14].

### Search Strategy

We conducted a comprehensive search on PubMed, Embase, Web of Science, Cochrane Library, ClinicalTrials, China National Knowledge Infrastructure, Wanfang, China Science and Technology Journal Database and SinoMed, searching for relevant human studies published from inception up to 12 August 2022. No restriction was made on the language of the papers identified. The search strategy is presented in appendix 1.

### Selection of studies

All titles and abstracts were independently screened by 2 reviewers (XL and LH). Any disagreements were resolved by a third reviewer (ZZ). The full text of selected studies was independently assessed for eligibility by 3 reviewers (XL, LH and ZZ), and any conflicts were resolved by discussion with the senior author (JC).

### Data extraction

A data extraction form was designed and approved by all the authors in advance. Two independent reviewers (XL and JT) independently screened the included articles and extract the data. A third reviewer (JM) checked all the extracted data. Disagreements regarding the selection and quality assessment of the articles were resolved through group discussion and full consensus.

### Quality Assessment

The Newcastle-Ottawa Scale was applied to evaluate the quality of each study, using the following 9-point scoring method: 4 points for population selection, 2 points for group comparison and 3 points for outcome [15]. Two review authors (XL and ZZ) independently assess the included articles. Any disagreements were resolved by discussion with the senior author (JC).

### Data Analysis

The primary outcome of the study was mortality after Fontan procedure, including all-cause mortality and post-heart transplant (HT) mortality. Meta-analysis was performed using Stata (version 17.0) and results were presented as odds ratios (OR) and 95% confidence interval (CI). The pooled OR was calculated using random effects (Mantel-Haenszel) models. The *I*^*2*^ statistic was used to assess heterogeneity between studies, and a value greater than 50% can be considered as having substantial heterogeneity [16]. Sensitivity analysis was performed to determine the potential influence of each study on the overall pooled results. Subgroup analysis and meta-regression analysis were conducted to explore the potential sources of heterogeneity. A *p*-value less than 0.05 was considered statistically significant.

## Results

### General Study characteristics

**Fig. 1:**
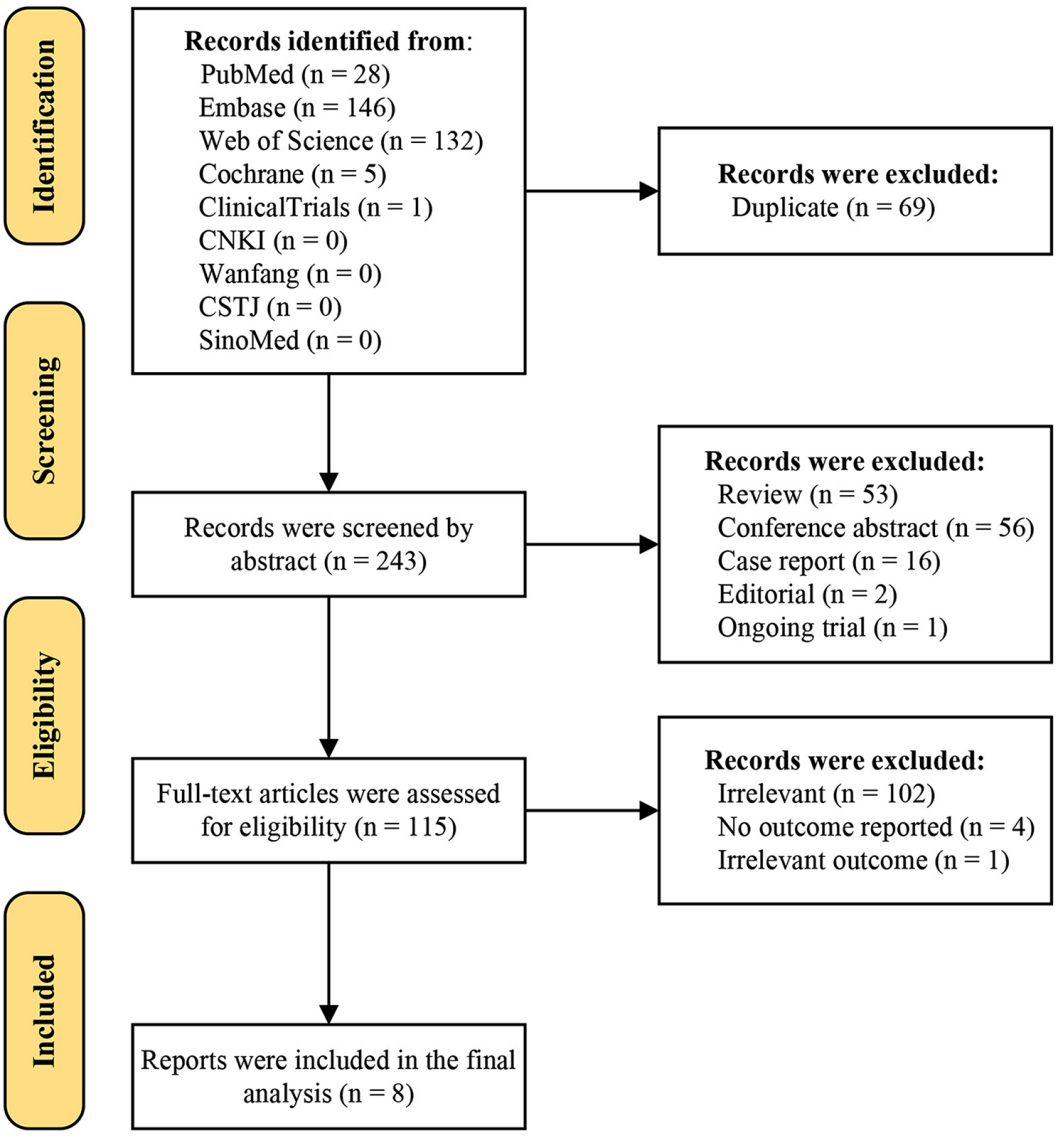
PRISMA flow diagram of study selection

Flow chart according to PRISMA is presented in Fig. 1. A total of 312 studies were initially identified, of which 69 were excluded due to duplication. Then another 128 records were removed after title and abstract screening, and there were 115 papers for full paper review. Finally, eight studies were included for statistical synthesis, comprising 2,466 patients in total. The median number of patients in each study was 115 (range 20 − 1,436), with a median follow-up of 4.8 years (range 1.0-28.5 years). The characteristics of each study are summarized in Table 1 and the quality assessments are presented in Supplementary Table 1.

**Table 1:** Study characteristics of the included articles

First author	Year	Journal	Country	Center	Study type	Data collection period	Follow-up duration	Included patients	LD	Deaths	Determination of LD	Comparation
Emamaullee	2022	*Ann Surg*	USA	Multiple	ROS	1992–2018	12.6 (8.4, 17.3) years	1436	225	96	Codes for cirrhosis/fibrosis	Non-LD vs. LD
Pundi	2016	*Am J Cardiol*	USA	Single	ROS	1973–2012	20.6 ± 8.1 years	195	40	45	Histopathology/imaging	Non-cirrhotic vs. Cirrhotic
Emamaullee	2021	*JHEP Rep*	USA	Single	ROS	2011–2020	median of 2 years	106	37	7	Histopathology	CHFS 0-2b vs. CHFS 3–4
Elder	2015	*Congenit Heart Dis*	USA	Single	ROS	1999–2013	28.5 ± 7.7 years	123	21	13	Imaging	VAS score 0–1 vs. VAS score 2–3
Sganga	2021	*J Heart Lung Transplant*	USA	Single	ROS	2006–2019	17 (4, 49) months	32	10	7	Histopathology/imaging	Non-cirrhotic vs. Cirrhotic
Amdani	2022	*J Thorac Cardiovasc Surg*	USA	Multiple	ROS	2005–2018	NR	524	132	93	MELD-XI score	MELD-XI score < 11.5 vs. MELD-XI score ≥ 11.5
Hofferberth	2017	*Pediatr Transplant*	USA	Single	ROS	1988–2015	median of 4.8 years	30	22	9	Histopathology/imaging	Normal vs. Abnormal liver findings
Simpson	2014	*J Heart Lung Transplant*	USA	Single	ROS	2004–2012	1 year	20	7	5	Histopathology/imaging	Non-cirrhotic vs. Cirrhotic

LD, liver disease; ROS, retrospective observational study; CHFS, congestive hepatic fibrosis score; VAS score, varices, ascites, splenomegaly; MELD-XI, model for end-stage liver disease excluding international normalized ratio; NR, not reported

### Overall analysis

**Fig. 2:**
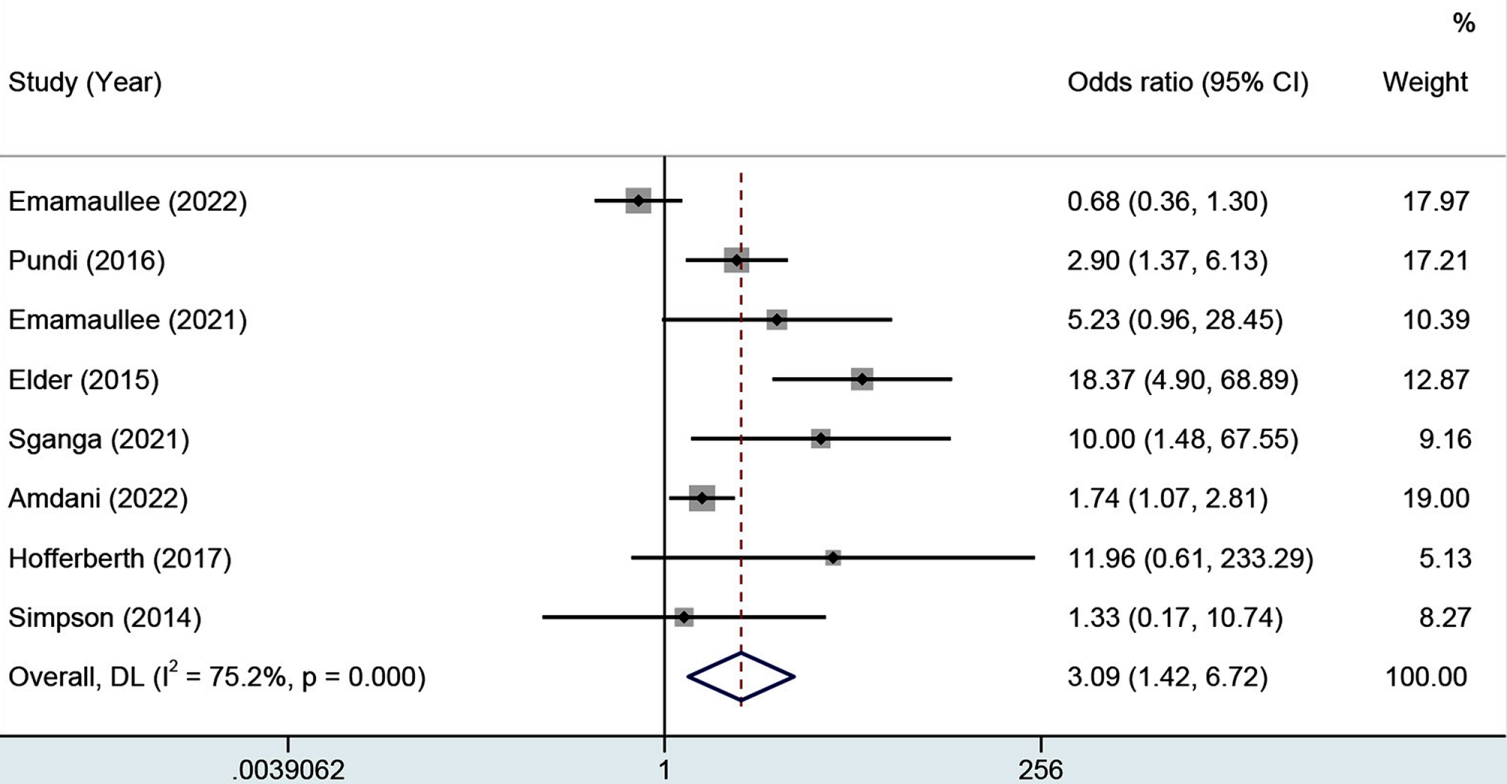
Forest plot demonstrating the influences of liver disease on overall mortality of Fontan patients. A total of 2,466 patients from 8 studies were included. The meta-analysis yielded an OR of 3.09 with a 95% CI of 1.42–6.72, with significant heterogeneity identified (*I*^*2*^ = 75.2%, *p* < 0.001). OR, odds ratios; CI, confidence interval

Overall, a total of 2,466 patients undergoing the Fontan procedure, from 8 studies [7–11, 13, 17, 18], were included in this analysis. There were 275 deaths in total and yielded a summary mortality of 11.15%. The overall mortality of patients with more severe liver disease was 18.02%, while for those with minor or absent liver damage, it was 9.43%. The meta-analysis yielded an OR of 3.09 with a 95% CI of 1.42–6.72, as shown in Fig. 2. However, the ORs varied substantially among individual studies (range 0.68–18.37) and the outcomes were highly heterogeneous across studies (*I*^*2*^ = 75.2%, *p* < 0.001).

### Sensitivity analysis

Influence Analysis (metan-based) was performed to determine the influence of individual studies on the pooled OR. As shown in Table 2, the pooled effect size was stable when given named study was omitted, which indicated the robustness of the meta-analysis estimate.

**Table 2:** **Sensitivity analysis of the pooled results (given named study was omitted)**

Study omitted	Estimate	95% conf. interval	
Emamaullee (2022)	4.1107178	1.9372162	8.7228266
Pundi (2016)	3.2809782	1.2708867	8.4703205
Emamaullee (2021)	2.9225979	1.2693291	6.7292072
Elder (2015)	2.1759692	1.1066173	4.2786625
Sganga (2021)	2.7273842	1.2234879	6.0798513
Amdani (2022)	3.7937186	1.3080765	11.002644
Hofferberth (2017)	2.8690000	1.2928351	6.3667523
Simpson (2014)	3.3783616	1.4646288	7.7926416
Combined	3.0877830	1.4196725	6.7159178

### Subgroup analysis

**Fig. 3:**
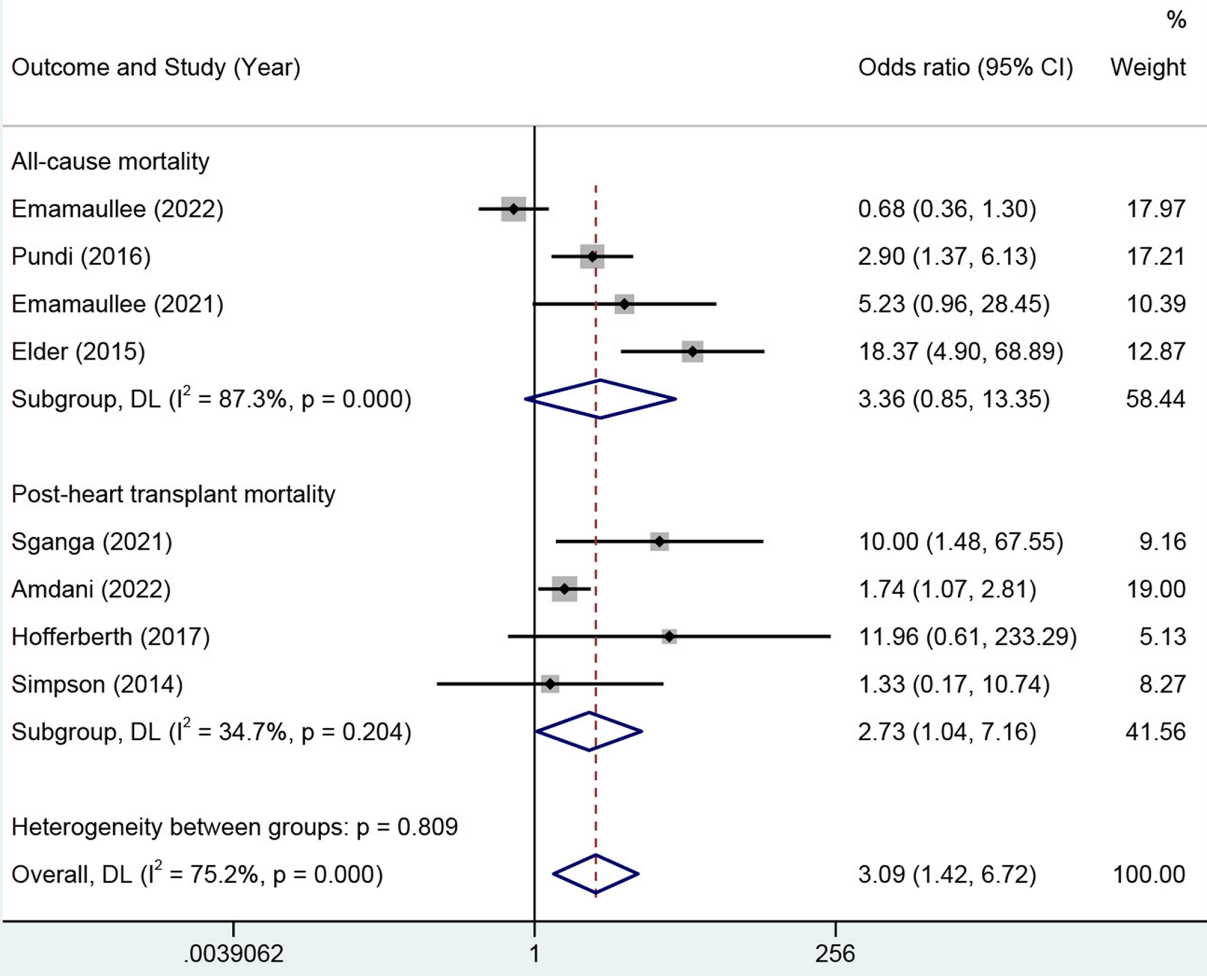
Forest plot of the subgroup analysis by outcomes. A total of 1,860 patients from 4 studies were included in the analysis of all-cause mortality. The meta-analysis yielded an OR of 3.36 with a 95% CI of 0.85–13.35, with considerable heterogeneity observed (*I*^*2*^ = 87.3%, *p* < 0.001). A total of 606 patients from 4 studies were included in this analysis of post-heart transplant. The meta-analysis yielded an OR of 2.73 with a 95% CI of 1.04–7.16, with a significant decline in heterogeneity (*I*^*2*^ = 34.7%, *p* = 0.204). OR, odds ratios; CI, confidence interval

Subgroup analysis stratified by all-cause mortality and post-HT mortality was also conducted, as shown in Fig. 3. A total of 1,860 patients, from 4 studies [11, 13, 17, 18], were included in the analysis of all-cause mortality. There were 161 deaths in total and yielded a summary mortality of 8.66%. The overall mortality of patients with more severe liver disease was 12.69%, while for those with minor or absent liver damage, it was 7.81%. The meta-analysis yielded an OR of 3.36 with a 95% CI of 0.85–13.35. The ORs varied substantially among individual studies (range 0.68–18.37) and considerable heterogeneity across studies was still observed (*I*^*2*^ = 87.3%, *p* < 0.001). A total of 606 patients, from 4 studies [7–10], were included in this analysis of post-HT mortality. There were 114 deaths and yielded a summary mortality of 18.81%. The overall mortality of patients with more severe liver disease was 28.07%, while for those with minor or absent liver damage, it was 15.17%. The meta-analysis yielded an OR of 2.73 with a 95% CI of 1.04–7.16. The results exhibited a relatively narrow range of ORs (range 1.33–11.96), and the heterogeneity was significantly reduced (*I*^*2*^ = 34.7%, *p* = 0.204).

### Meta-regression

To further explore the potential heterogeneity sources, meta-regression was conducted to investigate the impact of diagnostic methods for liver disease on the primary outcome. The diagnostic methods in the included studies could be stratified into histopathology/imaging, imaging alone, blood test alone and international classification of disease (ICD) codes. The results of meta-regression suggested that, compared to ICD codes, liver damage determined by histopathology/imaging or imaging alone had significant influences on the mortality after Fontan procedure, with a 95% CI of 1.48–18.03 and 3.36-216.66, respectively (Table 3).

**Table 3:** Meta-regression on diagnostic methods for liver disease

logor	exp(b)	Std. err.	t	*P*>|t|	95% conf. interval	
Histopathology/imaging	5.158169	2.325067	3.64	0.022	1.475633	18.03071
Imaging alone	26.985410	20.245950	4.39	0.012	3.361049	216.6622
Blood test alone	2.550056	1.048006	2.28	0.085	0.8147039	7.981779
_cons	0.680924	0.224017	-1.17	0.308	0.2731513	1.697436

### Re-analysis after excluding FALD patients identified by ICD codes

**Fig. 4:**
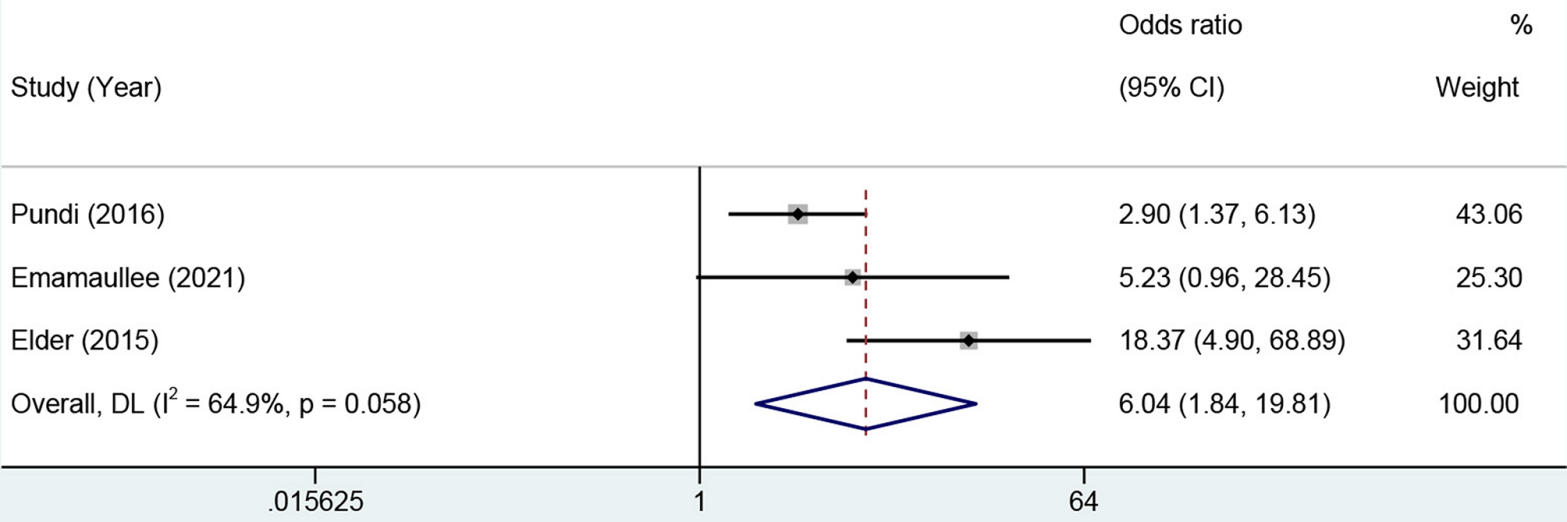
Forest plot of pooled OR for all-cause mortality excluding FALD patients identified by ICD codes. A total of 424 patients from 3 studies were included. The meta-analysis yielded an OR of 6.04 with a 95% CI of 1.84–19.81, with an obvious reduction in heterogeneity (*I*^*2*^ = 64.9%, *p* = 0.058). OR, odds ratios; CI, confidence interval

Our meta-regression analysis showed that diagnostic methods may be one source of the heterogeneity. In the study conducted by Emamaullee et al., presumed FALD was identified by ICD codes, which was thought to result in an underestimation of the complications [17]. Therefore, it was excluded from the subsequent analysis (Fig. 4). A total of 424 patients, from 3 studies [11, 13, 18], were included in the analysis of all-cause mortality. There were 65 deaths in total and yielded a summary mortality of 15.33%. The overall mortality of patients with more severe liver disease was 30.61%, while for those with minor or absent liver damage, it was 10.74%. The meta-analysis yielded an OR of 6.04 with a 95% CI of 1.84–19.81. Furthermore, the heterogeneity was obviously declined (*I*^*2*^ = 64.9%, *p* = 0.058).

## Discussion

The discrepancies between studies reported on the mortality of Fontan patients with liver disease make it important to integrate these information from different studies. This current meta-analysis, inclusive of 2,466 Fontan patients from 8 studies, revealed a significant association between death after Fontan procedure and the severity of liver disease, which was confirmed by sensitivity analysis and subgroup analysis assessing post-HT mortality. Meta-regression showed that diagnostic methods for liver disease may be a source of heterogeneity. After removal of the FALD patients identified by ICD codes, heterogeneity was markedly reduced, and the positive association between all-cause mortality and the severity of liver disease became significant. To our knowledge, this is the first systematic review and meta-analysis evaluating the influences of FALD on the mortality of this complex population.

### The pathogenesis of FALD and diagnostic considerations

It has been suggested that risk factors such as pre-Fontan hypoxemia, perioperative liver injury, high central venous pressures and reduced hepatic blood flow may contribute to FALD [19]. Additionally, early diagnosis of FALD is very difficult due to the lack of typical signs and symptoms [19], which may lead to an underestimate of this disease [17]. Liver biopsy is considered the gold standard for diagnosing FALD and is now recommended for routine monitoring for patients undergoing Fontan operation more than 10 years [6], but it is an invasive procedure and the potential complications limit its routine use in clinical practice. Therefore, imaging examination and blood test remain the main alternatives for assessing liver disease in this population, despite the diagnosis value remains controversial. It was found that there is no significant correlation between the severity of architectural changes on liver biopsy and laboratory results or imaging findings [20]. However, another research showed that histologic fibrosis has a strong correlation with magnetic resonance elastography [21]. And model for end-stage liver disease excluding international normalized ratio (MELD-XI) scores were found to be significantly associated with hepatic fibrosis scores [22].

### The effects of FALD on mortality

The incidence of FALD after Fontan palliation is very high, and virtually all patients have developed fibrosis by adolescence [6]. However, the prognostic significance of liver disease in Fontan patients remains controversial [7–13]. Recent studies have revealed that late referral after Fontan failure and markers of failing Fontan physiology (worse functional status, lower extremity varicosities and venovenous collaterals) were associated with post-transplant mortality [23], but not fibrosis/severe liver abnormalities on liver imaging [9, 24]. Wu et al. [12] found that the degree of hepatic fibrosis fails to predict transplant-free survival or overall survival. Also, patients with cirrhosis identified by CT have similar post-HT mortality compared to those without cirrhosis [10]. By contrast, it was observed that bridging fibrosis is associated with worse survival in these patients [11]. And among HT patients, cirrhosis on pre-transplant imaging is associated with worse outcomes [7]. In addition, portal hypertension was proved to be significantly predictive of adverse events (death, cardiac transplantation, or listing) [13], and higher MELD-XI scores at HT has been shown to portend poorer post-HT survival [8]. Notably, our meta-regression analysis clearly revealed that liver disease determined by histopathology/imaging or imaging alone affect the overall mortality in a significant manner. And the pooled estimate was stable and was not affected when individual studies were separately omitted. These results on the one hand explains the source of heterogeneity, and on the other hand it suggests the potential role of imaging examinations in routine surveillance of liver disease.

### The sources of heterogeneity

Of note, substantial heterogeneity between studies was observed, which may be explained by the primary outcome measure, diagnostic methods for liver disease, single-center experiences and relatively small sample sizes. These factors may account for the divergent findings across studies and bias our meta-analysis results. Therefore, subgroup analysis was also conducted and the results confirmed a significant positive correlation between the severity of liver disease and post-HT mortality. Meanwhile, heterogeneity was obviously reduced after the studies assessing all-cause mortality were removed, which indicated that findings related to post-HT mortality were consistent across these studies included. And after excluding the patients identified by ICD codes, we found a significant positive association between the severity of liver disease and all-cause mortality, and furthermore, the heterogeneity was markedly decreased. The results showed that diagnostic methods for liver disease might be the primary source of heterogeneity.

### The therapeutic considerations

Unfortunately, Fontan patients often suffer from multiple organ dysfunction and poor long-term outcomes and impaired quality of life. It was found that more than half of the failing Fontan patients were treated with pulmonary vasodilators [25]. And in another study, bosentan was showed to decrease postexercise heart rate and improve New York Heart Association Functional Class and 6-minute walking test results in Fontan patients [26]. However, for patients with failing Fontan circulation and advanced liver disease, the only effective treatment may be heart transplant, alone or combined with liver transplantation. The latest research demonstrated that combined heart-liver transplant probably confers a survival benefit for Fontan patients with advanced liver disease [7, 17, 27]. Consistently, our results suggested that combined heart-liver transplantation may be a better choice for such patients.

### Limitations

The present study has several limitations. First, the included studies were retrospective case series or cohort studies, they are subject to inherent biases and confounders. Secondly, the follow-up duration varied across the studies. A longer duration of follow-up is necessary to assess the influences of liver disease on mortality. Thirdly, considerable heterogeneity across studies was observed in the overall analysis and subgroup analysis for all-cause mortality. Finally, identification of liver disease, grouping methods and comparisons varied among different studies.

## Conclusions

Our present meta-analysis revealed a significant association between the severity of liver disease following Fontan procedure and mortality. Therefore, lifelong follow-up is required and imaging examinations are recommended for surveillance of liver disease. Among patients with failing Fontan circulation and advanced liver disease, combined heart-liver transplantation may provide additional survival benefits.

### Electronic supplementary material

Below is the link to the electronic supplementary material.

Supplementary Material 1

Supplementary Material 2

## Data Availability

The datasets used and/or analysed during the current study are available from the corresponding author on reasonable request.

## Abbreviations

FALD: Fontan-associated liver disease
PRISMA: Preferred Reporting Items for Systematic reviews and Meta-Analyses
HT: Heart transplant
OR: Odds ratios
CI: Confidence interval
ICD: International classification of disease
MELD-XI: Model for end-stage liver disease excluding international normalized ratio (MELD-XI)
