# Supplementary material for: Effect of liver abnormalities on mortality in Fontan patients: a systematic review and meta-analysis
Source: BMC Cardiovasc Disord. 2024 Jul 25;24:385. doi: 10.1186/s12872-024-04042-3 (PMC11270789; doi:10.1186/s12872-024-04042-3)
Supplement: Supplementary file 1 — Supplementary Material 1 [file 12872_2024_4042_MOESM1_ESM.docx]

**Supplementary Table 1. Quality assessment of the included studies.**

| Study | Representativeness of the exposed cohort | Selection of the non exposed cohort | Ascertainment of exposure | Demonstration that outcome of interest was not present at start of study | Comparability of cohorts on the basis of the design or analysis | Assessment of outcome | Was follow-up long enough for outcomes to occur | Adequacy of follow up of cohorts | Quality score |
| --- | --- | --- | --- | --- | --- | --- | --- | --- | --- |
| Emamaullee 2022 | 1 | 1 | 1 | 1 | 1 | 1 | 1 | 0 | 7 |
| Pundi 2016 | 1 | 1 | 1 | 1 | 2 | 1 | 1 | 0 | 8 |
| Emamaullee 2021 | 1 | 1 | 1 | 1 | 2 | 1 | 1 | 0 | 8 |
| Elder 2015 | 1 | 1 | 1 | 1 | 1 | 1 | 1 | 0 | 7 |
| Sganga 2021 | 1 | 1 | 1 | 1 | 2 | 1 | 1 | 0 | 8 |
| Amdani 2022 | 1 | 1 | 1 | 1 | 1 | 1 | 1 | 0 | 7 |
| Hofferberth 2017 | 1 | 1 | 1 | 1 | 2 | 1 | 1 | 0 | 8 |
| Simpson 2014 | 1 | 1 | 1 | 1 | 2 | 1 | 1 | 0 | 8 |
