# Supplementary material for: Effect of liver abnormalities on mortality in Fontan patients: a systematic review and meta-analysis
Source: BMC Cardiovasc Disord. 2024 Jul 25;24:385. doi: 10.1186/s12872-024-04042-3 (PMC11270789; doi:10.1186/s12872-024-04042-3)
Supplement: Supplementary file 2 — Supplementary Material 2 [file 12872_2024_4042_MOESM2_ESM.docx]

**Search Strategy**

(((((((((((fontan procedure[MeSH Terms]) OR (fontan operation[Title/Abstract])) OR (fontan palliation[Title/Abstract])) OR (fontan circulation[Title/Abstract])) OR (fontan circuit[Title/Abstract])) OR (stage III norwood procedure[Title/Abstract])) OR (atriopulmonary connection[Title/Abstract])) OR (right atrium–right ventricular connection[Title/Abstract])) OR (total cavopulmonary connection[Title/Abstract])) AND (((((((((liver diseases[MeSH Terms]) OR (liver disease[Title/Abstract])) OR (liver dysfunction[Title/Abstract])) OR (liver dysfunctions[Title/Abstract])) OR (liver cirrhosis[Title/Abstract])) OR (hepatic cirrhosis[Title/Abstract])) OR (liver fibrosis[Title/Abstract]))) OR (hepatic fibrosis[Title/Abstract]))) AND (((((((mortality[MeSH Terms]) OR (mortalities[Title/Abstract])) OR (fatality rate[Title/Abstract])) OR (fatality rates[Title/Abstract])) OR (death rate[Title/Abstract])) OR (death rates[Title/Abstract])) OR (survival[Title/Abstract]))) AND ("cohort studies"[mesh] OR "case-control studies"[mesh] OR "comparative study"[pt] OR "risk factors"[mesh] OR "cohort"[tw] OR "compared"[tw] OR "groups"[tw] OR "case control"[tw] OR "multivariate"[tw])
